# Metabolic Capability of a Predominant *Halanaerobium* sp. in Hydraulically Fractured Gas Wells and Its Implication in Pipeline Corrosion

**DOI:** 10.3389/fmicb.2016.00988

**Published:** 2016-06-22

**Authors:** Renxing Liang, Irene A. Davidova, Christopher R. Marks, Blake W. Stamps, Brian H. Harriman, Bradley S. Stevenson, Kathleen E. Duncan, Joseph M. Suflita

**Affiliations:** Department of Microbiology and Plant Biology and OU Biocorrosion Center, University of OklahomaNorman, OK, USA

**Keywords:** halophilic, *Halanaerobium*, thiosulfate reducing bacteria, guar gum, biocorrosion, hydraulic fracturing

## Abstract

Microbial activity associated with produced water from hydraulic fracturing operations can lead to gas souring and corrosion of carbon-steel equipment. We examined the microbial ecology of produced water and the prospective role of the prevalent microorganisms in corrosion in a gas production field in the Barnett Shale. The microbial community was mainly composed of halophilic, sulfidogenic bacteria within the order *Halanaerobiales*, which reflected the geochemical conditions of highly saline water containing sulfur species (S_2_O_3_^2-^, SO_4_^2-^, and HS^-^). A predominant, halophilic bacterium (strain DL-01) was subsequently isolated and identified as belonging to the genus *Halanaerobium*. The isolate could degrade guar gum, a polysaccharide polymer used in fracture fluids, to produce acetate and sulfide in a 10% NaCl medium at 37°C when thiosulfate was available. To mitigate potential deleterious effects of sulfide and acetate, a quaternary ammonium compound was found to be an efficient biocide in inhibiting the growth and metabolic activity of strain DL-01 relative to glutaraldehyde and tetrakis (hydroxymethyl) phosphonium sulfate. Collectively, our findings suggest that predominant halophiles associated with unconventional shale gas extraction could proliferate and produce sulfide and acetate from the metabolism of polysaccharides used in hydraulic fracturing fluids. These metabolic products might be returned to the surface and transported in pipelines to cause pitting corrosion in downstream infrastructure.

## Introduction

It is estimated that the percentage of total natural gas supplies from geological shale formations will increase from approximately 20% (2010) to 50% by 2025 in the United States (Davis et al., 2012). Such remarkable growth of shale gas production is largely attributed to the advancement of hydraulic fracturing technologies (Vengosh et al., 2014). Hydraulic fracturing involves the process of injecting large volumes of water, proppant and a myriad of chemical additives into the deep subsurface with high pressure to liberate natural gas from shale formations (Gregory et al., 2011). Microbial activity (e.g., biogenic sulfide production) associated with produced water can lead to gas souring and corrosion of production facilities (Davis et al., 2012). Since biocorrosion can lead to disruptions of gas pipelines and storage tanks, considerable efforts have been made by the oil and gas industry to monitor and control deleterious microbial activities during hydraulic fracturing operations (Struchtemeyer et al., 2012; Gaspar et al., 2014; Kahrilas et al., 2014).

Despite treatments of the fracturing fluid with biocides and the extreme physicochemical conditions (e.g., high salinity, Supplementary Table S1) in the fractured deep subsurface, a stream of recent studies (see Supplementary Table S1) has revealed that similar dominant microorganisms were associated with produced water from various hydraulically fractured shale formations in the United States (Davis et al., 2012; Struchtemeyer and Elshahed, 2012; Murali Mohan et al., 2013a; Strong et al., 2013; Wuchter et al., 2013; Cluff et al., 2014). Typically, the microbial community was mostly composed of halophiles including acid-producing bacteria and multiple lineages of sulfidogenic microorganisms (Davis et al., 2012; Struchtemeyer and Elshahed, 2012; Murali Mohan et al., 2013a,b; Cluff et al., 2014). Most of the dominant halophilic species were affiliated with the genus *Halanaerobium*, which can sometimes account for over 99% of the microflora in produced water (Murali Mohan et al., 2013a; Cluff et al., 2014). Members of the genus *Halanaerobium* are well known for fermentation of carbohydrates and sulfide production through thiosulfate reduction (Ravot et al., 1997, 2005). Therefore, *Halanaerobium* is clearly an important genus that might play vital roles in the biodegradation of organic matter and production of sulfide in fractured shale formations (Akob et al., 2015).

Numerous organic chemical additives are typically found in hydraulic fracturing fluids (Stringfellow et al., 2014; Kekacs et al., 2015) that could serve as electron donors to support microbial growth and sulfidogenic processes (Youssef et al., 2009; Strong et al., 2013). Among them, guar gum is a principal component of fracturing fluids and is commonly used as a thickening agent in the oil and gas industry (Lester et al., 2013; Stringfellow et al., 2014). Recently, activated sludge and microbial mats were proposed as efficient biological treatments to aerobically degrade such high-molecular weight polysaccharides in flowback wastewater from hydraulic fracturing (Lester et al., 2013; Akyon et al., 2015). Additionally, other studies have indicated that concentrations of dissolved organic carbon in produced water decreased rapidly via abiotic or biotic processes in the subsurface (Strong et al., 2013; Cluff et al., 2014). However, the biodegradation of guar gum by the dominant anaerobic microorganisms in the fractured shale formation remains unknown. In this regard, we hypothesized that the dominant *Halanaerobium* species in the produced water can contribute to the decomposition of guar gum and the production of acetate and sulfide in the fractured deep subsurface.

Better understanding of the *in situ* metabolic activity of the prevalent *Halanaerobium* in complex subterranean environments may help in the control of these acid-producing and sulfidogenic organisms and further mitigate biocorrosion problems. However, culture-independent molecular approaches only provide phylogenetic information and genomic potential of the associated microbial community (Mohan et al., 2014). These molecular techniques such as high-throughput sequencing typically cannot distinguish between DNA from live organisms and dead cells (Cluff et al., 2014), potentially leading to biased conclusions on the *in situ* metabolic activity of indigenous microorganisms. In contrast, cultivation-based approaches can provide complementary insight on the physiology and possible interactions among the live, cultivable microbes. The efficacy of biocides against the indigenous microorganisms can also be critically evaluated against isolates or enrichment cultures to provide guidance on field treatment (Struchtemeyer et al., 2012; Gaspar et al., 2014; Santillan et al., 2015). Therefore, the integration of both culture-dependent and -independent techniques will help to gain a better understanding of the fate of organic matter in the fractured deep subsurface and the potential role of associated metabolites such as organic acids and sulfide in the corrosion of carbon-steel pipelines.

The objective of this study was to examine the microbial ecology of produced water associated with hydraulic fracturing and the potential roles of the dominant microorganisms in corrosion of gas pipelines. We characterized the geochemistry and microbial assemblages in produced water from a hydraulically fractured site in the Barnett Shale (Texas City, Texas, USA). One of the predominant organisms (*Halanaerobium* sp. strain DL-01) was subsequently isolated and its metabolic capability in the biodegradation of guar gum was further examined under both fermentative and thiosulfate-reducing conditions. Moreover, the efficacy of biocides against strain DL-01 was critically evaluated to potentially decrease the prevalence of *Halanaerobium* and thereby mitigate deleterious biocorrosion processes in shale gas production facilities.

## Materials and Methods

### Site Description and Samples Collection

**FIGURE 1:**
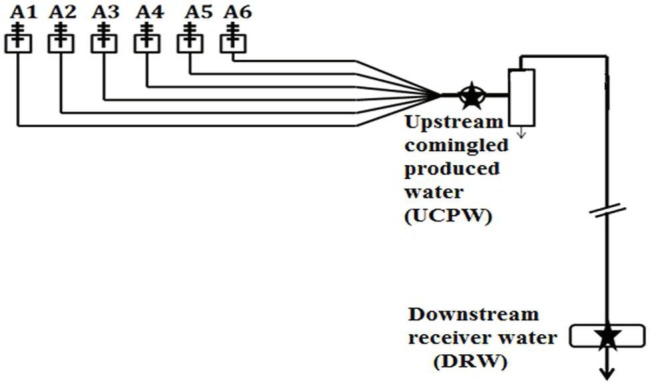
**Schematic diagram of the shale gas production facility in Barnett Shale.** Six wells (A1–A6) were drilled in this site and the stars indicated the locations where upstream (UCPW) and downstream (DRW) samples were collected. The distance between the two locations is around 1 km. Severe corrosion was detected in the downstream pipeline where replacement was mandated for maintenance.

Samples of produced water were collected from a shale gas production field in the Barnett Shale near Arlington, Texas, USA in July and September 2012, respectively. Multiple wells (A1–A6) were hydraulically fractured in the sampling site and the schematic is represented in **Figure 1**. The upstream comingled produced water (UCPW) near the gas-water separator (**Figure 1**) was collected in a 1L polypropylene bottle closed without a headspace (Nalgene, Rochester, NY, USA). The temperature of the upstream sample was ~37°C. Downstream water from the receiver (DRW) along the gas pipeline (**Figure 1**) was obtained in the similar manner. The samples for geochemical analyses and cultivation were stored at 4°C before use. For microbial community characterization, 250–300 mL samples were filtered in the field using Nalgene analytical filter funnels containing nitrocellulose filters (sterile, 0.45 μm, Nalgene Fisher Scientific, Pittsburgh, PA, USA). The filters were placed into sterile 50 mL centrifuge tubes and preserved with 1 mL of DNAzol (DNAzol DN127, Molecular Research Center, Inc., Cincinnati, OH, USA). The tubes containing the filters were placed on ice until returned to the laboratory (within 5 h) and then transferred to a freezer (-85°C) until DNA extraction.

### Microbial Enumeration

A most probable number (MPN) procedure was used to enumerate sulfate-reducing (SRB), acid-producing (APB), and thiosulfate-reducing bacteria (TRB). For SRB and APB, commercially produced media (C&S Labs, Tulsa, OK, USA) were directly compared with media prepared in the lab upon return of samples. All MPN media formulations were adjusted to pH 7.0 and salinities of 5% and 10% (w/v) NaCl, based on available commercial medium salinities routinely employed at this site. The enumeration of SRB was conducted using RST-API medium (Tanner, 1989). The media for APB and TRB enumeration were prepared according to NACE (National Association of Corrosion Engineers) standard TM0194 and others (TM0194, 2004; Tanner et al., 2007). The original aqueous sample (1 mL) was inoculated into 9 mL of medium in Balch tubes with a sterile syringe flushed with N_2_. Serial dilutions (10-fold) were made by transferring 1 mL of each dilution to separate Balch tubes containing 9 mL of fresh medium. MPN tubes were prepared in triplicate and incubated at 37°C for 4 weeks. Each group of bacteria was enumerated with the statistical table described previously (Banwart, 1981).

### Microbial Community Characterization

Filters preserved in the field were thawed and DNA was extracted using the Promega Maxwell^®^ 16 Tissue LEV Total RNA purification kit as described previously (Oldham et al., 2012). The pooled 16S rRNA gene library was prepared with primers S-D-Arch-0519-a-S-15 and S-D-Bact-0785-b-A-18 (Klindworth et al., 2012) modified to include a 16 bp M13 sequence to allow for the addition of a 12 bp barcode in a two-step PCR unique to each library (Hamady et al., 2008; Wawrik et al., 2012). The libraries were sequenced on the Illumina MiSeq platform using V2 PE250 chemistry. Paired reads were joined and then demultiplexed in QIIME (Quantitative Insights Into Microbial Ecology) software package (Caporaso et al., 2010). Chimeras were removed and Operational Taxonomic Units (OTUs) were assigned at 97% similarity using USEARCH 6.1.544 (Edgar, 2010). Taxonomy was assigned using the RDP (Ribosomal Database Project) naive Bayesian classifier (Wang et al., 2007) against the SILVA (r SSU, small subunit) database release 111 (Yilmaz et al., 2013). Raw sequences were submitted to the NCBI sequence read archive (SRA) database (Accession number: SRX1046644).

### Enrichment and Isolation

Samples (10 mL) of upstream, comingled water (UCPW) and downstream water from the receiver (DRW) were inoculated into 160 mL serum bottles containing 50 mL of reduced marine mineral medium (Widdel and Bak, 1992) adjusted to 4 and 10% salinity. In order to test potential relevant electron donors in the fractured subterranean systems, the incubations received the following compounds as sources of energy and carbon: (1) gas condensate (1 μL); (2) 20 psi gas mixture of methane, ethane, butane, and propane (1:1:1:1); (3) 20 psi H_2_:CO_2_ (80:20); (4) a mixture of pyruvate (10 mM) and lactate (10 mM); (5) elemental iron (as granules). Sterile and substrate-free controls were provided. All enrichments were incubated at 31°C and monitored for growth by following sulfate consumption and microscopic cell counts. Incubations showing active growth were transferred, and enrichments obtained in 10% NaCl medium were used for isolations.

Initial isolations were performed using serial 10-fold dilutions in the same basal medium, (Widdel and Bak, 1992) containing 10% NaCl and glucose (20 mM) as a growth substrate at 31°C. After several subsequent dilutions, pre-purified cultures were used as inoculum for anaerobic culturing bottles containing the same glucose-based medium solidified with 2% agar. After 7 days of incubation, isolated colonies were picked from the culture bottles inside an anaerobic glove chamber. An isolate obtained in this manner and designated strain DL-1 was maintained in sulfate-free, reduced marine mineral medium containing 10% NaCl, glucose (20 mM) and yeast extract (0.001%). The purity of the isolate was checked by microscopy. Growth was tested with the following electron donors: galactose (20 mM), mannose (20 mM), guar gum (0.5% w/v), and cellulose (0. 2% w/v cut filter paper). Thiosulfate (10 mM) and sulfate (20 mM) were tested as electron acceptors. Growth was monitored by optical density measured at 600 nm (where possible), protein production and by sulfide production (see Analytic techniques).

### Sequencing and Phylogenetic Analysis

Cells in stationary phase cells were collected by centrifugation at 8000 × *g* for 15 min at room temperature. Cell pellets were further treated with 20 μg/mL proteinase K (Promega) for 15 min at room temperature. Genomic DNA was extracted with the Maxwell 16 Tissue LEV total RNA purification kit as previously described (Oldham et al., 2012). The 16S rRNA gene of strain DL-01 was amplified using PCR Supermix (Invitrogen, Carlsbad, CA, USA) with universal primers fD1 and rP2 described previously (Weisburg et al., 1991). The sequence was determined by Sanger sequencing on ABI 3730 (Applied Biosystems, Foster City, CA, USA). The quality of the obtained sequence was verified and assembled with the program suite Sequencher version 5.1 (Gene Codes Corp., Ann Arbor, MI, USA). The assembled sequence (length = 1362 bp) was aligned with other closely related type strain sequences retrieved from the NCBI Genbank. The phylogenetic analysis was performed using MEGA 6.0 using the neighbor-joining method (Tamura et al., 2013) and bootstrap analysis with 1000 replicates. The GenBank/EMBL/DDBJ accession number for the 16S rRNA gene sequence of strain DL-01 is KR612329.

### Analytical Techniques

The anions sulfate and chloride were analyzed by ion chromatography as described previously (Lyles et al., 2013). Sulfate was determined in the samples pretreated using Dionex OnGuardII Ag/Na cartridges (Thermo Fisher Scientific, Sunnyvale, CA, USA) to remove halides as described by the manufacturer. The pH was measured in the field with pH strips (color pHast indicator strips pH 5–10; EM Science, Gibbstown, NJ, made in Germany). The concentration of thiosulfate was quantified using an iodometric CHEMetrics thiosulfate titration kit (CHEMetrics, Inc., Calverton, VA, USA). Dissolved ferrous iron and sulfide were measured by the ferrozine assay (Stookey, 1970) and methylene blue method (Tanner, 1989) as previously described. Protein was determined using the Thermo Scientific^TM^ Pierce^TM^ BCA^TM^ Protein Assay (Thermo Scientific, Pittsburgh, PA, USA) according to manufacturer’s instructions. Fermentation products such as formate, acetate, pyruvate, and lactate were measured by high performance liquid chromatography (HPLC, Dionex model IC-3000, Sunnyvale, CA, USA) as previously described. (Lyles et al., 2014) The wavelength of the UV absorbance detector was set at 210 nm and the mobile phase was 60% (vol/vol) KH_2_PO_4_ (25 mM, pH 2.5) and 40% acetonitrile. The pump was operated at a flow rate of 1 mL/min. In addition, portions of the samples were diluted in 30 mM oxalic acid in order to measure ethanol by gas chromatography with flame ionization detection (GC-FID) under the operating conditions described in Davidova et al. (2012).

### Efficacy of Biocides against Strain DL-01

The efficacy of common biocides used in the oil and gas industry was determined against *Halanaerobium* sp. strain DL-01 under fermentative and thiosulfate-reducing conditions. The biocides tested included glutaraldehyde, tetrakis (hydroxymethyl) phosphonium sulfate (THPS) and benzyldodecyldimethylammonium chloride (a representative quaternary ammonium compound, abbreviated as QAC). Glucose (20 mM) was used as a substrate and strain DL-01 was grown in 10% NaCl marine mineral medium (Widdel and Bak, 1992) at 37°C. The assay was performed in Balch tubes containing 9 mL medium and 1 mL cells of strain DL-01. The inoculated media were exposed to different dosages of biocides (final concentrations varying from 0 mg/L to 500 mg/L depending on the minimum inhibitory concentration of each biocide) under fermentative conditions (no electron acceptor) and thiosulfate-reducing conditions (10 mM thiosulfate). The corresponding sterile (inoculated with heat-killed cells of strain DL-01) and inhibitor-free controls served as the basis for determining the efficacy of biocides. Optical density (600 nm wavelength) was measured over time to monitor the microbial growth. Additionally, acetate and sulfide were measured at the conclusion of the experiment as further evidence of microbial activity.

## Results

### Geochemistry and Microbial Enumerations

The geochemistry of the produced water in the upstream (UCPW) and downstream (DRW) samples is summarized in **Table 1**. The pH in all samples was between 6.5 and 7.0. The content of Cl^-^ in the UCPW (4.68–11.7%) was much higher than the DRW sample (1.69–1.97%). The low salinity in the downstream produced water might be attributed to the introduction of external water into the system. Notably, dissolved ferrous iron (Fe^2+^) in DRW (11.76 mM) was much higher than UCPW (0.73 mM) probably due to the corrosion that occurred in the downstream pipeline (DRW) (**Figure 1**). In addition to sulfate and sulfide, a low level of thiosulfate was detected in the DRW (0.17 ± 0.01 mM). These data suggest that sulfate and thiosulfate could be potential electron acceptors for sulfide production. Interestingly, the concentration of acetate in the DRW (170 mM) was much higher than the UCPW sample (0.5 mM). The number of cultivable acid producing bacteria in UCPW was 5 × 10^3^ cells/mL when grown in medium containing 10% NaCl (Supplementary Table S2), which was close to the salinity of the original sample (11.7% NaCl). However, only minimal growth of thiosulfate and sulfate-reducing bacteria was observed. None of the tubes in the DRW sample showed growth.

**Table 1:** Geochemical characteristics of produced water from the upstream (UCPW) and downstream (DRW) of a shale gas production facility in Barnett Shale formations (**Figure 1**).

Samples	Sampling time	pH	Sulfate (mM)	Fe^2+^ (mM)	Sulfide (mM)	Thiosulfate (mM)	Salinity (Cl^-^ g/L)	Acetate (mM)
Upstream(UPCW)	July 2012	7.0	0.74	1.73	0.04	BDL^a^	117	NR^b^
	September2012	6.8	1.84	0.73	BDL	BDL	46.8	0.5
Downstream(DRW)	July 2012	6.5	0.79	NR	0.25	BDL	19.7	NR
	September2012	7.0	1.93	11.76	BDL	0.17	16.9	170

*^a^BDL, below detection level (5 ppm); ^b^NR, data not recorded.*

### Microbial Community Characterization

**FIGURE 2:**
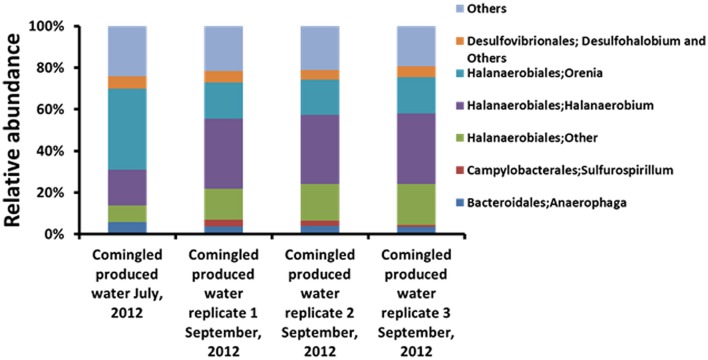
**Relative abundance of major taxa (Genus level classification) in upstream comingled produced water (UCPW).** Three biological replicates (1, 2, and 3) were included in the later produced water collected in September, 2012.

No quantifiable or amplifiable DNA could be extracted from DRW samples. In contrast, an appreciable amount of DNA was obtained from UCPW sampled in both July and September, 2012. The microbial assemblages associated with the UCPW samples were characterized by high-throughput sequencing of PCR-amplified 16S rRNA gene libraries. Sequences affiliated with the order Halanaerobiales were numerically dominant (64.4–70.7%) in both UCPW samples (**Figure 2** and Supplementary Figure S1). Representative sequences from these OTUs were primarily affiliated with the genera *Orenia* and *Halanaerobium* (**Figure 2**). Interestingly, the relative abundance of OTUs most closely related to the genus *Halanaerobium* increased to become the most dominant taxon (from 17 to ~33%) in the produced water collected in September, whereas the genus *Orenia* decreased from 39 to ~17% (**Figure 2**). In addition, sequences affiliated with the order *Desulfovibrionales* only accounted for ~5% of the total bacterial community (Supplementary Figure S1).

### Isolation and Characterization

**FIGURE 3:**
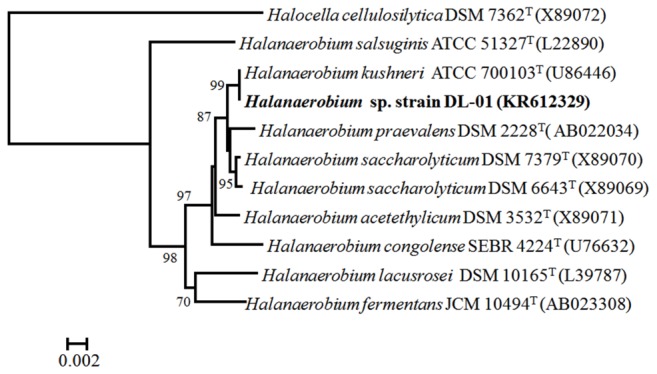
**Phylogenetic relationship of *Halanaerobium* sp. strain DL-01 (in bold) to other species within the genus *Halanaerobium*.** The accession number of each strain is indicated in the parenthesis. The tree was constructed based on approximately 1362 bp 16S rRNA gene sequences. One thousand bootstrap replications were performed and only those greater than 700 are shown. Bar indicated 2 nucleotide substitutions per 1000 bp.

Microbial growth was initially observed with the UCPW inoculum when either pyruvate or lactate served as a substrate. Subsequent transfers revealed that the enrichment was also capable of growth with glucose as a carbon and energy source. Repeated transfer of the enrichment culture and eventual isolation of individual colonies on the same glucose-based medium solidified with 2% agar ultimately was used to obtain a pure culture. The colonies were round, smooth and opaque. Cells of strain DL-01 were short rods and usually appeared in pairs or have been assembled in string-like chains (Supplementary Figure S2). Isolate DL-01 could grow under a broad range of salinities ranging from 2 to 15% NaCl, and could ferment glucose, galactose, mannose and the polysaccharide, guar gum. Thiosulfate was found to be a suitable electron acceptor when strain DL-01 was grown with various carbohydrates including guar gum. Sulfate was not utilized as an electron acceptor when strain DL-01 was grown with guar gum. Phylogenetic analysis based on 16S rRNA gene sequences indicated that strain DL-01 was likely a member of the genus *Halanaerobium* and most closely related to the type strain of *Halanaerobium kushneri* ATCC 700103^T^ (**Figure 3**). Notably, the 16S rRNA gene sequence of strain DL-01 places it within OTU-3 (Supplementary Figure S3), suggesting that the dominant *Halanerobium* phylotype was isolated from UCPW.

### Acetate and Sulfide Production by *Halanaerobium* sp. Strain DL-01

**FIGURE 4:**
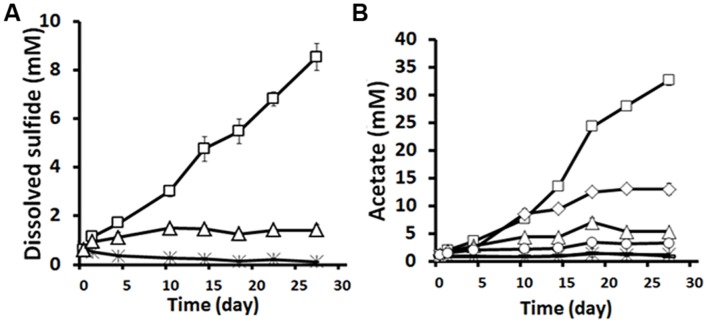
**Sulfide (A) and acetate (B) production by strain DL-01 when grown on 0.5% guar gum under fermentative and thiosulfate-reducing conditions.** Legends: □, 0.5% guar gum+thiosulfate; △,no guar gum+thiosulfate; 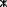, sterile (0.5% guar gum+thiosulfate); ◇, 0.5% guar gum+no thiosulfate; °, no guar gum+no thiosulfate; -, (overlapping data with 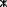), sterile (0.5% guar gum+ no thiosulfate). Sterile indicates that cells of DL-01 were heat killed by autoclaving.

Strain DL-01 was capable of growth with guar gum as the sole carbon and energy source under both fermentative and thiosulfate-reducing conditions. During the growth with guar gum and thiosulfate, 7.1 ± 0.3 mM dissolved sulfide (excluding background level) was accumulated in 27 days (**Figure 4A**). No significant sulfide production was detected in the sterile control and the control without guar gum. The growth with guar gum without an electron acceptor resulted in accumulation of acetate (11.8 ± 0.8 mM, **Figure 4B**). However, if thiosulfate was provided as an electron acceptor, twice the amount of acetate was produced during the same time period (**Figure 4B**). In addition, small amounts of formate (0.2–0.9 mM) and ethanol (1.1–3.4 mM) were also detected.

### Efficacy of Biocides against Strain DL-01

**FIGURE 5:**
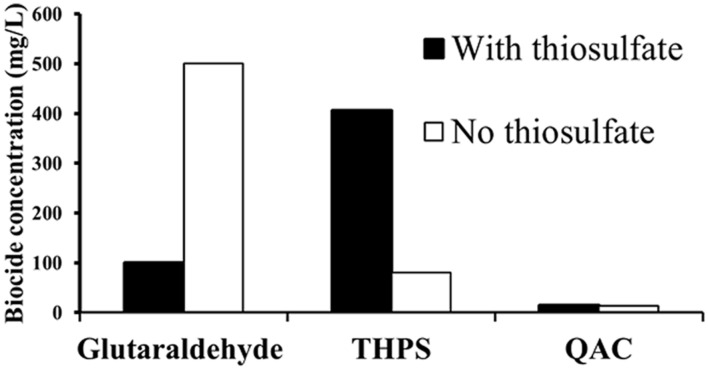
**Minimum inhibitory concentrations of biocides against *Halanaerobium* DL-01 based on microbial growth, sulfide, and acetate production relative to sterile controls, which the cells of DL-01 were heat-killed by autoclaving.** Black and clear bars indicate the conditions with and without thiosulfate, respectively.

The efficacy of biocides against strain DL-01was assessed on the basis of sulfide and acetate production (Supplementary Figures S4 and S5) and growth relative to sterile controls. A relatively low dosage of QAC (13.5 mg/L) was sufficient to completely inhibit acetate and sulfide production under both thiosulfate-reducing (Supplementary Figure S4B) and fermentative conditions (Supplementary Figure S4A). The efficacy of glutaraldehyde against strain DL-01 was much higher in the presence of thiosulfate (100 mg/L, Supplementary Figures S5A,B) than in its absence (500 mg/L, Supplementary Figure S4B). In contrast, THPS (81 mg/L) completely inhibited microbial growth and thus, acetate production when no thiosulfate was present (Supplementary Figure S5D). However, up to 406 mg/L THPS showed no inhibition on sulfide and acetate production when thiosulfate was present (Supplementary Figures S5C,D). Accordingly, the minimum inhibitory concentrations (the lowest dosage to completely inhibit microbial growth and activity) of the three biocides are summarized in **Figure 5**. The minimum inhibitory concentration of QAC (13.5 mg/L) was much lower than that of glutaraldehyde (500 mg/L in the absence of thiosulfate) and THPS (no inhibition up to 406 mg/L in the presence of thiosulfate).

## Discussion

Microbial activities associated with hydraulically fractured shale formations are of great concern to the oil and gas industry due to the potential for corrosion of pipelines, separators and storage tanks (Struchtemeyer and Elshahed, 2012). We characterized the geochemistry and microbial community of produced water from a shale gas production field in part of the Barnett Shale system. Typically, low levels of acetate (up to ~0.9 mM) are detected in produced waters from hydraulically fractured sites (Orem et al., 2014). In contrast, we detected an extremely high concentration of acetate (170 mM) in DRW (**Figure 1**). While acetate can be introduced through hydraulic fracturing (Sydansk, 1988), microbial activity might play a substantial role in the production of acetate. Regardless of the sources of acetate, its accumulation in produced water can potentially exacerbate corrosion of carbon-steel equipment (Azambuja and Muller, 1994; Suflita et al., 2008). In addition, sulfidogenic activity might also contribute to the observed corrosion of carbon-steel in the DRW where sulfide (0.25 mM) and sulfate (0.74–1.93 mM) were detected. Although the rapid rate of microbial metabolism (Jørgensen, 1990) makes measurement of thiosulfate quite challenging, thiosulfate (0.17 ± 0.01 mM) was detected in the DRW produced water when measured in the field using a thiosulfate titration kit. Moreover, a recent study found that the major fraction of the total sulfur in produced water was present as non-sulfate compounds and thereby the importance of sulfidogenic potential of non-sulfate-reducing microorganisms was implied in the fractured subsurface (Murali Mohan et al., 2013a). In fact, fermentative, thiosulfate-reducing bacteria have been demonstrated to be important in catalyzing corrosion of carbon steel in oil production facilities (Magot et al., 1997; Liang et al., 2014).

Molecular characterization of the microbial assemblages in each sample revealed that the most abundant taxa in the upstream produced water were members of the order *Halanaerobiales*, which is consistent with previous studies on the microbial ecology of produced water from shale gas extraction (Davis et al., 2012; Struchtemeyer and Elshahed, 2012; Murali Mohan et al., 2013a,b; Strong et al., 2013; Wuchter et al., 2013; Cluff et al., 2014). Such predominance of halophilic microorganisms suggested that the relative high salinity in the upstream produced water plays important roles in shaping the microbial community. Earlier studies have shown that members of the genus *Halanaerobium* increased dramatically over time in the produced water from the Marcellus and Barnett shale formations in the United States (Davis et al., 2012; Murali Mohan et al., 2013a; Cluff et al., 2014). Despite the limited sampling time points in this study (July and September, 2012), the relative abundance of *Halanaerobium* increased from 17 to ~33% in the later produced water sample (**Figure 2**). Although sulfate-reducing bacteria within *Deltaproteobacteria* were not typically detected in previous studies (Murali Mohan et al., 2013a; Cluff et al., 2014), members of the order *Desulfovibrionales* were present in low relative abundance (~5%; Supplementary Figure S1). The genera *Desulfohalobium* (Ollivier et al., 1991; Jakobsen et al., 2006) and *Desulfovermiculus* (Belyakova et al., 2006) were reported to be halophilic sulfate reducers, suggesting that sulfate reduction might also contribute to sulfidogenesis in subterranean systems with high salinity. Sequences affiliated with *Epsilonproteobacteria* (~3%) were detected in the produced water sampled in September. The majority of the OTUs within the *Epsilonproteobacteria* were affiliated with the genus *Sulfurospirillum* (**Figure 2**), a group of metabolically versatile bacteria previously shown to reduce sulfur and thiosulfate (Kodama and Watanabe, 2007). The presence of multiple lineages of sulfidogenic organisms and sulfur species (S_2_O_3_^2-^, SO_4_^2-^, and HS^-^) suggested that active sulfur cycle involving thiosulfate might be occurring in the deep subterranean shale formations after hydraulic fracturing.

The ubiquity and abundance of members of the genus *Halanaerobium* found in high salinity production water suggests that they could play crucial roles in the cycling of carbon and sulfur in these environments (Murali Mohan et al., 2013b; Cluff et al., 2014). Cultivation of the abundant microbes in produced water is important to understand the physiology of these taxa and their potential role in corrosion. A numerically dominant organism was isolated from the produced water in this study and identified as *Halanaerobium* sp. DL-01. This isolate was highly similar to the most abundant OTU from the genus *Halanaerobium* within the microbial assemblage of upstream produced water (Supplementary Figure S3). Further phylogenetic analysis revealed that strain DL-01 was most closely related to *H. kushneri* isolated from high saline produced water from an oil reservoir in Central Oklahoma (Bhupathiraju et al., 1999). *H. congolense* (Ravot et al., 1997) and *H. salsugo* (Bhupathiraju et al., 1999) have also been isolated from oil fields, alluding to the ecological importance of the genus *Halanaerobium* in high saline produced water in oil and gas production facilities.

Many species of *Halanaerobium* are capable of fermenting carbohydrates to acetate and other organic acids (Ravot et al., 1997, 2005). We found that strain DL-01 could produce acetate through the fermentation of guar gum, the major gelling agent in the fracturing fluid to increase viscosity (Lester et al., 2013). Most importantly, strain DL-01 was able to generate sulfide from the reduction of thiosulfate, but it was unable to utilize sulfate. Thiosulfate and elemental sulfur may be used as electron acceptors by various *Halanaerobium* species but none of the validated described organisms are known to use sulfate in this manner (Oren, 2015). Of the numerous chemical additives introduced into the shale formation during hydraulic fracturing, the organic constituents in the fracturing fluid, such as hydrocarbon distillates and carbohydrate polymers (e.g., guar gum) (Cluff et al., 2014), could conceivably serve as electron donors to stimulate potentially deleterious microbial processes such as acetate and sulfide production. The fate of the organic matter in fracturing fluids injected into the deep subsurface is poorly understood, but a recent metagenomic study of produced water from shale gas extraction revealed a relatively high abundance of functional genes associated with metabolism of mono- and polysaccharides (Mohan et al., 2014). Furthermore, other studies have found that the concentration of dissolved organic matter in produced water decreased over time due to abiotic or biotic processes occurring in the deep subsurface (Cluff et al., 2014; Orem et al., 2014). Until the study described here, the numerically dominant *Halanaerobium* spp. in produced water had not been shown to biodegrade organic matter (e.g., guar gum) in the high saline water and contribute to the production of acetate and sulfide.

Since the corrosiveness of sulfide and acetate is well-known in the oil and gas industry (Liang et al., 2014), the collective findings led us to propose a possible corrosion scenario (Supplementary Figure S6) in the hydraulically fractured site in Barnett Shale. Fracturing fluids containing biodegradable polysaccharide polymers like guar gum (0.1–0.5%) get injected deep into shale formations (Lester et al., 2013). The abundant members of the genus *Halanaerobium* decomposed the guar gum, producing acetate, and sulfide (if thiosulfate is available). Eventually, the produced acetate and sulfide could be returned in the produced water and transported to downstream pipeline networks (Supplementary Figure S6). Acetate (170 mM) and sulfide (0.25 mM) detected in saline production waters could act synergistically (Singer et al., 2007) to corrode the downstream production facilities such as gathering pipelines and storage tanks.

To mitigate the potential corrosion caused by the abundant members of the genus *Halanaerobium*, the efficacy of three biocides was evaluated against *Halanaerobium* sp. DL-01. A relatively high dosage of glutaraldehyde (500 mg/L) was required to completely inhibit strain DL-01. This concentration was much higher than the minimum inhibitory concentrations for *Desulfovibrio alaskensis* strain G20 (12.5 mg/L) and a sulfate-reducing enrichment culture (100 mg/L) obtained from fracturing fluid (Struchtemeyer et al., 2012). Interestingly, exposure of *Pseudomonas fluorescens* to produced water can cause an increased resistance to glutaraldehyde (Vikram et al., 2014). It is unclear, however, whether the enhanced tolerance against glutaraldehyde in strain DL-01 was triggered by a similar mechanism. The presence of thiosulfate did increase the resistance of *Halanaerobium* DL-01 to THPS (no inhibition up to 406 mg/L). The ineffectiveness of THPS might be attributed to potential interaction between THPS and thiosulfate (Williams and McGinley, 2010). Notably, QAC was found to be more efficient than glutaraldehyde and THPS (**Figure 5**) under both thiosulfate-reducing and fermentative growth conditions. Therefore, the preferential utilization of QAC might be considered to decrease the microbial activity of the dominant *Halanaerobium* in high salinity produced water. In addition, future work is needed to assess the synergistic effect of multiple biocides (Kahrilas et al., 2014) against strain DL-01 and the underlying mechanisms for the potential resistance to glutaraldehyde and THPS in high saline brines (Vikram et al., 2014).

It is generally believed that problematic microorganisms are directly associated with severe corroding sites when assessing risks of biocorrosion in the oil and gas industry. The research presented here implicated that the sulfidogenic microorganisms (e.g., *Halanaerobium* sp. DL-01) in the produced water after hydraulic fracturing could play an important role in the biodegradation of organic carbon such as guar gum to produce acetate and sulfide in the fractured deep shale formations. The produced acetate and sulfide could be transported to aboveground with the returning water (Supplementary Figure S6) and might greatly contribute to corrosion of carbon-steel gathering pipelines and other equipment in distal, downstream locations (Supplementary Figure S6). The findings on the efficacy of biocides against strain DL-01 should ultimately help select suitable biocides to decrease the prevalence of *Halanaerobium* spp. in the shale formations and thereby mitigate detrimental biocorriosion processes during hydraulic fracturing operations (Struchtemeyer et al., 2012).

## Author Contributions

RL spearheaded the work under the supervision of JS. RL also drafted the manuscript with JS responsible for overall interpretation, coordination and editing of the paper. ID contributed to the isolation of the culture. CM and BH helped on the sampling and geochemical analyses. BWS, BS, and KD performed and helped interpret the molecular data in the manuscript. All authors reviewed the manuscript and contributed to the final revision of the manuscript.

## Conflict of Interest Statement

The authors declare that the research was conducted in the absence of any commercial or financial relationships that could be construed as a potential conflict of interest.
