# Supplementary material for: Metabolic Capability of a Predominant Halanaerobium sp. in Hydraulically Fractured Gas Wells and Its Implication in Pipeline Corrosion
Source: Front Microbiol. 2016 Jun 22;7:988. doi: 10.3389/fmicb.2016.00988 (PMC4916785; doi:10.3389/fmicb.2016.00988)
Supplement: Supplementary file 1 [file Presentation_1.PDF]

Supporting Information for:

Metabolic Capability of a Predominant *Halanaerobium* sp. in Hydraulically Fractured Gas Wells and its Implication in Pipeline Corrosion

Renxing Liang, Irene A. Davidova, Christopher R. Marks, Blake W. Stamps, Brian H. Harriman, Bradley S. Stevenson, Kathleen E. Duncan, Joseph M. Suflita*

Department of Microbiology and Plant Biology and OU Biocorrosion Center, University of Oklahoma, Norman, OK, USA

*To whom correspondence should be addressed. E-mail: jsuflita@ou.edu

Mailing address: 770 Van Vleet Oval, Norman, OK 73069, U.S.A.

Tel: +1(405) 325-3771; Fax: +1(405) 325-7619.

Supplementary Table

Table S1 Comparison of select geochemical characteristics and associated microflora in hydraulic fracturing operations.

Samples	Microbial community	Potential metabolic capability	Residence time in the formation	Salinity (mg/L)	pH	Location	Reference
Frac and flowback water	Distinctive communities between frac and flowback waters	Fermentation; sulfate reduction	24 hours – 2 months	458-2690	6.74-8.55	Barnett Shale	Struchtemeyer et al. 2011
Gas–water separator and water storage tank	Mainly halophilic members including the order Halanaerobiales	Fermentation; sulfate reduction; sulfur-reduction thiosulfate reduction	6 months	6000-10000	6.12-6.53	Barnett Shale	Davis, et al. 2012
Formation water from wells	Moderate halophiles within the Firmicutes and Methanogens	Fermentation; methanogenesis; anaerobic methane oxidation	5 months	55115-63145	6.56-6.97	Antrim Shale	Wuchter, et al. 2013
Flowback water impoundments	Bacterial communities in impoundments were depth dependent, but Halanaerobiales was most abundant	Fermentation; sulfate reduction; methanogenesis	N/A	32 100-56 800	6.5-7.2	Marcellus shale	Mohan et al. 2013a
Fracturing source water, fracturing fluid, and produced water	Increasing predominance of halotolerant anaerobes including Halanaerobiales	Fermentation; sulfate reduction; sulfur-reduction; thiosulfate reduction	1 to 187 days	3635-18 626	N/A	Marcellus shale	Mohan et al. 2013b
Flowback fluids and produced water from wellheads and gas-fluid separators	Dominance of *Halanerobium* in later produced water	Fermentation; sulfate reduction; thiosulfate reduction methanogenesis	0-328 days	10000-70000	5.2-6.3	Marcellus shale	Cluff, et al. 2014
Produced water from gas–liquid separator tank	Predominance of *Halanaerobium*	Fermentation; Sulfidogenesis; methanogenesis	5-38 months	109,000 - 184,000	N/A	Marcellus Shale	Akob et al. 2015

Table S2 Most probable number of of acid-producing bacteria, sulfate-reducing bacteria and thiosulfate-reducing bacteria in the upstream (UCPW) and downstream (DRW) samples

Bacterial groups	Salinity	Medium	UPCW	DRW
Sulfate-reducing bacteria	5% NaCl	C&S Labs	<1	0[a]
	10% NaCl	C&S Labs	0	0
Acid-producing bacteria	5% NaCl	C&S Labs	4	0
	10% NaCl	C&S Labs	$5x10^3$	0
Thiosulfate-reducing bacteria	5% NaCl	RST-TRB	2	0
	10% NaCl	RST-TRB	2	0

[a] indicates that no microbial activity was observed in any of the MPN tubes

Supplementary Figures

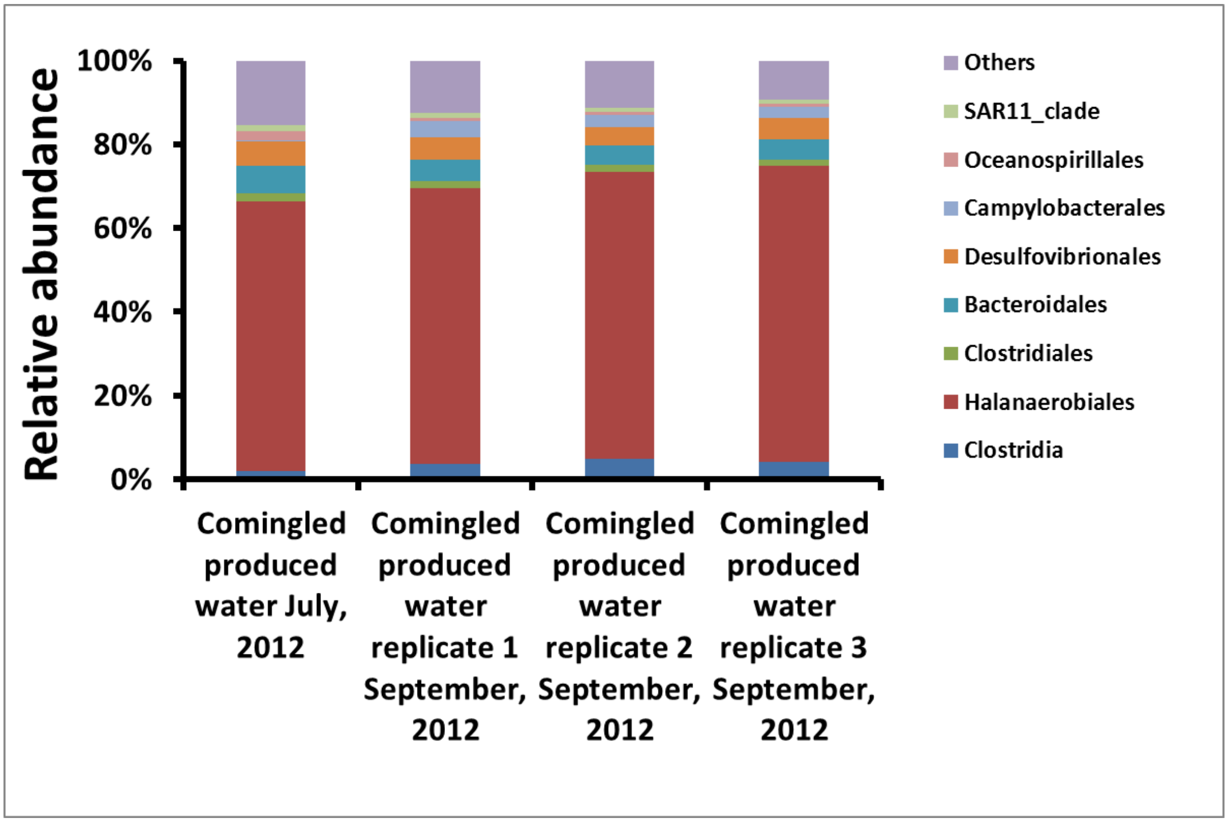

Figure S1. Relative abundance of major taxa (Order level) in upstream comingled production water. Three biological replicates (1, 2 and 3) were included in the later produced water collected in September, 2012.

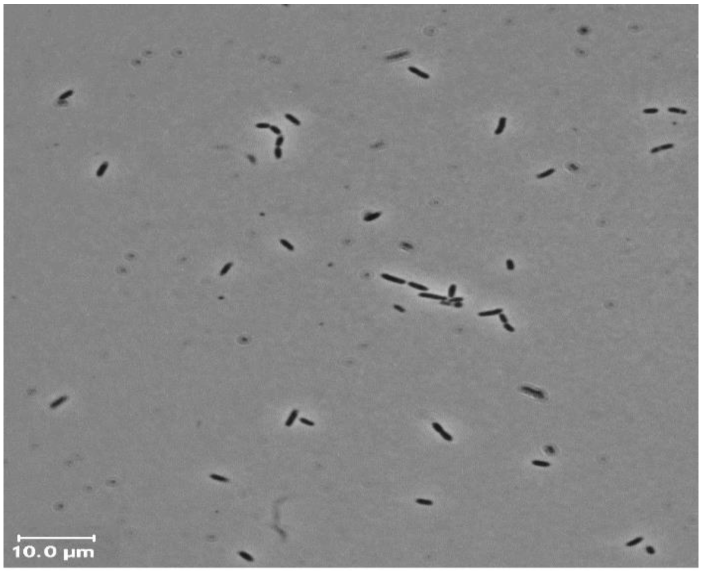

Figure S2. Cell morphology of *Halanaerobium* sp. DL-01. The image was taken under a Phase-contrast microscopy. Scale bar, 10 µm.

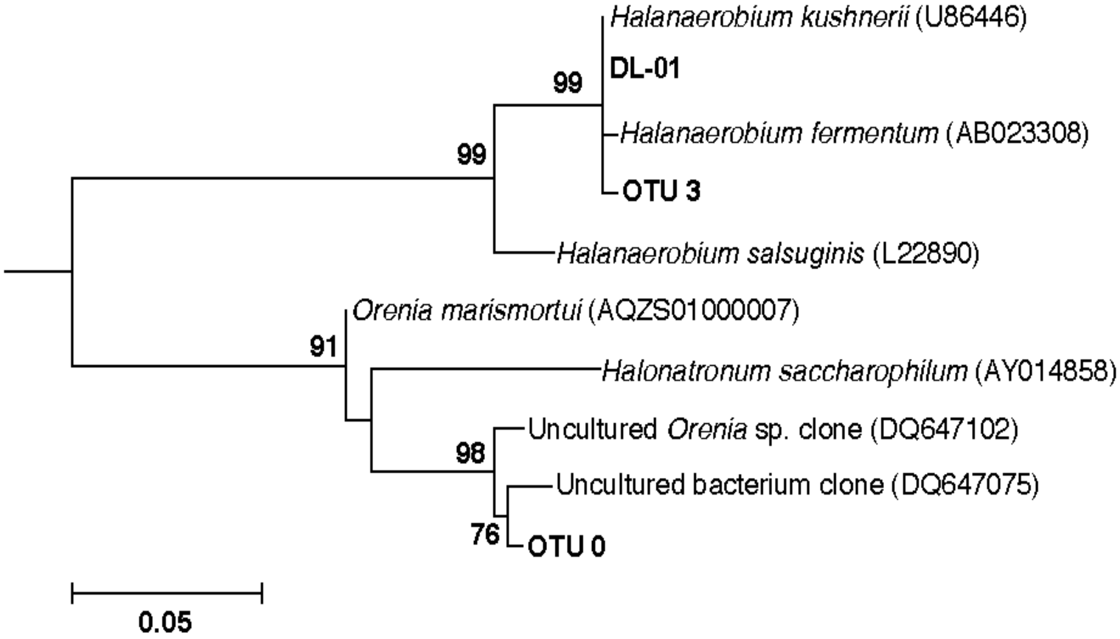

Figure S3. Phylogenetic relationship of *Halanaerobium* sp. DL-01 (in Bold) to other related species and the most related OTU (OTU 3 in bold) from the comingled produce water where DL-01 was originally isolated. The accession numbers are indicated in the parenthesis. Note: only a fragment (~300 bp) of 16S rRNA gene was used to construct the phylogenetic tree due to the limited sequence length from Illumina- based sequencing.

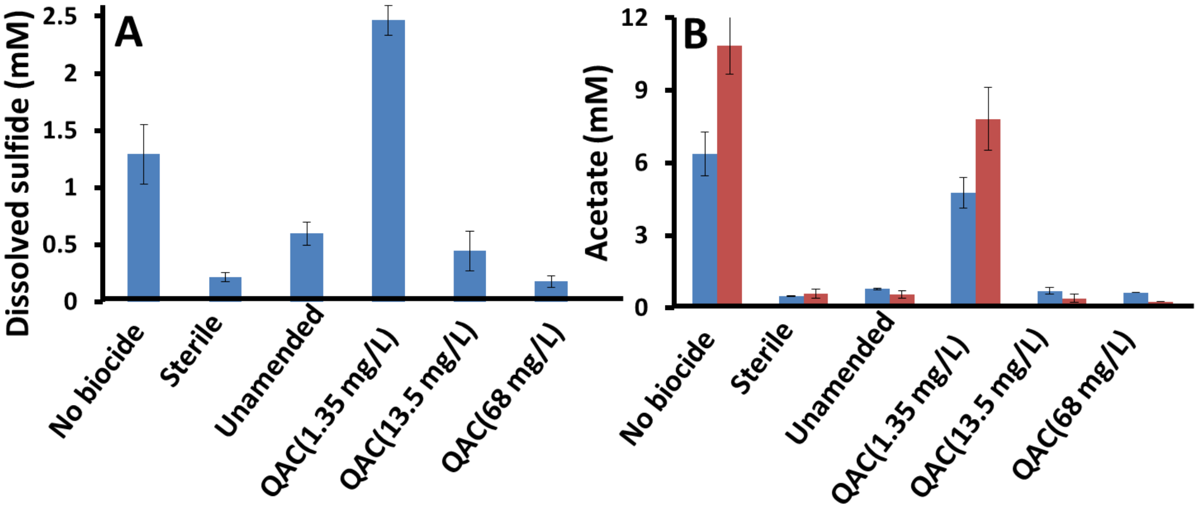

Figure S4. Efficacy of QAC on sulfide (A) and acetate (B) production by *Halanerobium* sp. DL-01. Blue bars indicate sulfide (A) or acetate (B) production under thiosulfate reducing conditions and red ones (B) refer to acetate production under fermentative conditions without thiosulfate. Note: 1.35, 13.5 and 68 mg/L correspond to 4, 40 and 200 μM, respectively.

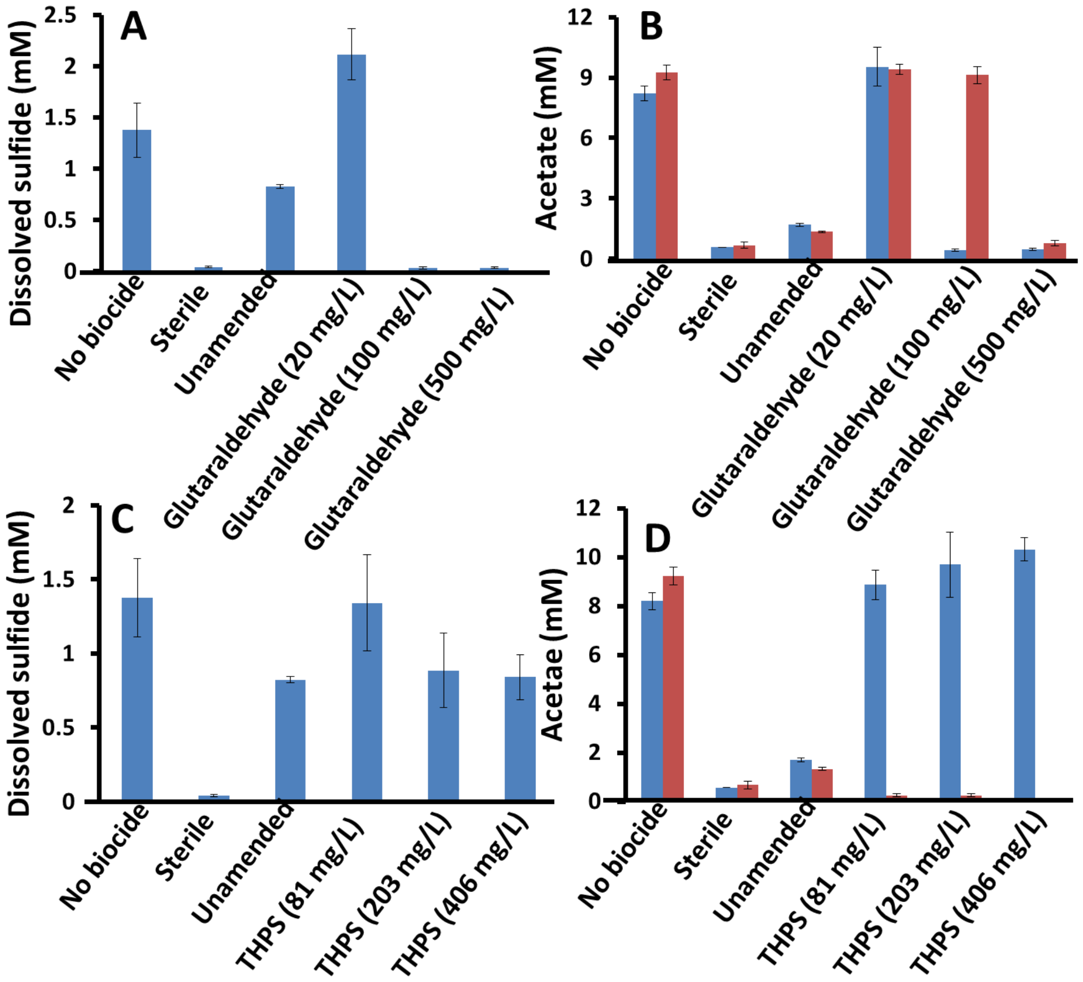

Figure S5. Efficacy of glutaraldehyde and THPS on sulfide (A & C) and acetate (B&D) production by *Halanerobium* sp. DL-01. Blue bars indicate sulfide (A & C) or acetate (B&D) production under thiosulfate reducing conditions and red ones (B&D) refer to acetate production under fermentative conditions without thiosulfate. Note: 20, 100 and 500 mg/L glutaraldehyde correspond to 200, 1000 and 5000 μM, respectively. 81, 203 and 406 mg/L THPS refer to 200, 500 and 1000 μM, respectively.

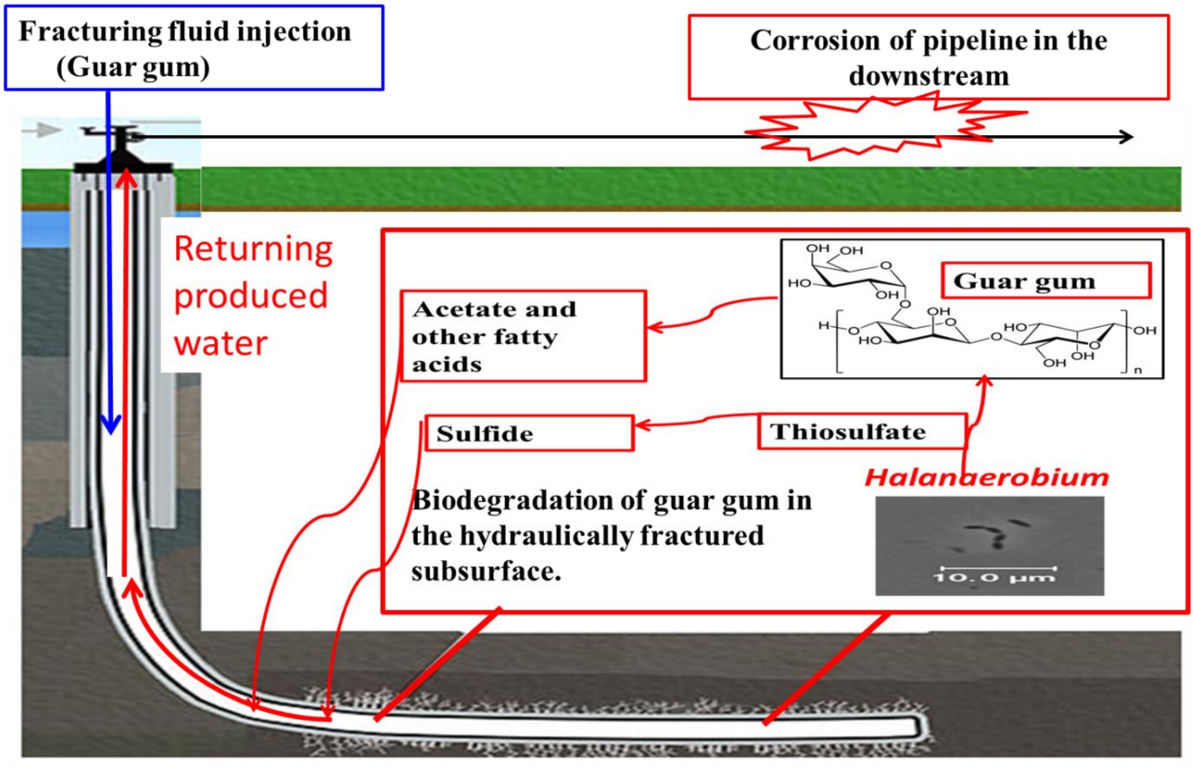

Figure S6. A proposed corrosion scenario in a hydraulically fractured site in Barnett Shale. Note: the schematic of the well was modified from http://www2.epa.gov/hfstudy.
